# Supplementary material for: Components of the Complement Cascade Differ in Polycystic Ovary Syndrome
Source: Int J Mol Sci. 2022 Oct 13;23(20):12232. doi: 10.3390/ijms232012232 (PMC9603248; doi:10.3390/ijms232012232)
Supplement: Supplementary file 1 [file ijms-23-12232-s001.zip › ijms-1940551-supplementary.pdf]

Supplementary figure S1.

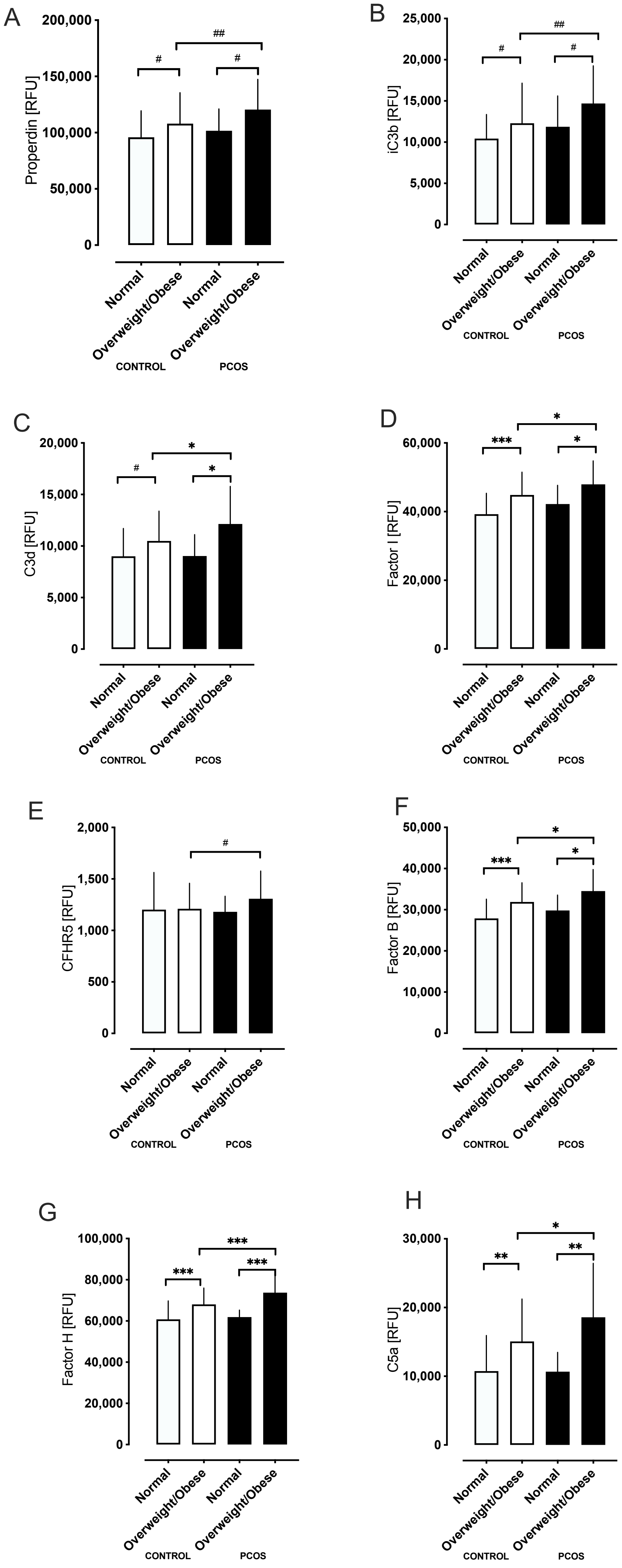

*Supplementary figure S1. Protein levels categorized by BMI for Properdin (A), iC3b (B), C3d (C), Factor I (D), CFHR5 (E), Factor B (F), Factor H (G) and C5a (H). When the control (white bars) and PCOS (black bars) subjects were categorized according to BMI into normal weight, defined as BMI <25, and overweight/obese, defined as BMI 25 or higher, the protein levels were still significantly higher in the overweight/obese PCOS women versus the overweight/obese control women. #p<0.05, ##p<0.005, *p<0.01, **p<0.001, ***p<0.0001.*
